# Measuring Marine Biotoxins in a Hypersaline Coastal Lagoon

**DOI:** 10.3390/toxins15090526

**Published:** 2023-08-26

**Authors:** Ainhoa Oller-Ruiz, Nuria Alcaraz-Oliver, Gema Férez, Javier Gilabert

**Affiliations:** Department of Chemical and Environmental Engineering, Technical University of Cartagena (UPCT), E-30203 Cartagena, Spain

**Keywords:** marine toxins, solid phase extraction, liquid chromatography, triple quadrupole mass spectrometry, coastal lagoons, Mar Menor

## Abstract

Marine biotoxins have posed a persistent problem along various coasts for many years. Coastal lagoons are ecosystems prone to phytoplankton blooms when altered by eutrophication. The Mar Menor is the largest hypersaline coastal lagoon in Europe. Sixteen marine toxins, including lipophilic toxins, yessotoxins, and domoic acid (DA), in seawater samples from the Mar Menor coastal lagoon were measured in one year. Only DA was detected in the range of 44.9–173.8 ng L^−1^. Environmental stressors and mechanisms controlling the presence of DA in the lagoon are discussed. As an enrichment and clean-up method, we employed solid phase extraction to filter and acidify 75 mL of the sample, followed by pre-concentration through a C18 SPE cartridge. The analytes were recovered in aqueous solutions and directly injected into the liquid chromatography system (LC-MS), which was equipped with a C18 column. The system operated in gradient mode, and we used tandem mass spectrometry (MS/MS) with a triple quadrupole (QqQ) in the multiple reaction monitoring mode (MRM) for analysis. The absence of matrix effects was checked and the limits of detection for most toxins were low, ranging from 0.05 to 91.2 ng L^−1^, depending on the compound. To validate the measurements, we performed recovery studies, falling in the range of 74–122%, with an intraday precision below 14.9% RSD.

## 1. Introduction

Certain marine phytoplankton species produce phycotoxins that are causing serious health problems for both humans, by triggering epidemic outbreaks, and ecosystems, by altering their structure. These toxins can bio-accumulate in the food chain [1,2,3,4,5], and humans can ingest them when they accumulate in filter feeders, such as shellfish or other seafood [6,7]. To ensure food safety, many countries’ food safety regulators have established maximum tolerable levels for these toxins in seafood [8,9,10], and as a result, monitoring and control programs for harmful algae blooms (HABs) are typically established in aquaculture and shellfish areas. However, the impact of HABs is not limited to human health only; it also affects the economy when mass fish mortality occurs in aquaculture areas or precautionary closures, also in touristic areas, are implemented. These events carry an annual estimated cost of 3–4 billion USD worldwide [11], with millions of dollars in one single event [12,13]. Harmful algal blooms have a significant impact not only on the aquaculture and fisheries sectors but also on the quality of recreational waters. The tourism industry, in particular, is highly vulnerable to this problem. Beach closures resulting from HABs, such as those that occurred between 2005 and 2009 in popular tourist destinations along the Mediterranean coasts of France and Italy [14,15,16,17,18] due to *Ostreopsis* outbreaks, caused significant economic losses. While most attention is focused on monitoring the effects of biotoxins in seafood, less attention is given to recreational waters where the quality of the bathing water is typically determined by the presence of fecal coliforms or intestinal enterococci only [19,20].

Although the food safety agencies have regulated the maximum levels of biotoxins in seafood, with lipophilic marine toxins (LMTs) mainly studied in contaminated bivalves, there is no regulation for toxin concentrations in bathing waters, and the levels of LMTs in seawater remain largely unexplored. Special attention has been paid to domoic acid, a potent amnesic toxin [21,22], which has been reported in the literature in several members of the marine food chain, from small organisms like copepods and krill to top predators such as seabirds and sea lions [23,24,25,26].

The objective of this study is to measure these toxins in a hypersaline coastal lagoon that is experiencing severe eutrophication to better understand the processes keeping them in the water. There are a few records of in-water marine biotoxins in Mediterranean coastal lagoons [27,28,29,30,31]. The Mar Menor is the largest hypersaline coastal lagoon in Europe, and is a popular tourist area, with large fisheries, and although it is not a shellfish cultivation area, cockles and sea snails are frequently consumed by local residents. To perform this study, we used solid phase extraction (SPE) coupled to liquid chromatography (LC)–tandem mass spectrometry (MS/MS) with a triple quadrupole (QqQ) in multiple reaction monitoring (MRM) mode. Based on existing methods, we adjusted different parameters to optimize the measurement of sixteen marine toxins in seawater, including lipophilic marine toxins, yessotoxins, and domoic acid (DA) in the Mar Menor coastal lagoon for the first time over the course of one year. However, the only recorded toxin in the water was DA at trace (ng·L^−1^) level.

The use of LC–MS methodologies for the detection of phycotoxins in these types of waters is currently on the rise as an alternative strategy to determine the presence of toxins and their possible implications in the ecosystem [32,33,34,35,36]. However, the analysis of in-water toxins using LC-MS systems requires pre-treatment of samples, including desalting and clean-up steps, as well as preconcentration techniques such as solid phase extraction (SPE) to achieve detection at low levels [1,37,38,39,40,41,42].

## 2. Results and Discussion

### 2.1. Enhancements in the LC-MS Analytical Procedure

To separate the toxins, a C18 stationary phase was utilized with a mixture of 2 mM ammonium acetate and 0.1% FA in water and MeOH as the mobile phase, based on the literature [27,32,38]. Three LC methods were explored using Zorbax SB-C18 column for DA, YTXs, and the remaining lipophilic toxins.

The percentage of MeOH in the mobile phase was examined to elute DA through the column. After selecting 25% MeOH, the percentage was increased to 95% to clean the column after each injection, and then the initial conditions were restored.

The separation of the remaining toxins was optimized by changing the percentage of MeOH at different times to achieve good resolution of all compounds in a shorter method. Temperature also affected the peak shapes, and experiments conducted in the range of 20–50 °C indicated that peaks approached each other as the temperature increased. Therefore, the temperature was adjusted to 30 °C to maintain constant column conditions.

The retention times (tR) for all compounds, along with their multiple reaction monitoring (MRM) transitions, are listed in Table 1. The MS/MS parameters were adopted from previous works [27,32,38]. YTX and hYTX detection was included and showed maximum sensitivity in negative ion mode (ESI-) with the deprotonated molecular ion [M-H]^−^.

### 2.2. Enhancements in the SPE Procedure

A silica C18 stationary phase, as in the LC column, was used for the SPE cartridge, which allowed analytes to be retained from a large volume sample and preconcentrated in a small elution fraction. To optimize the procedure, a 2 ng/mL aqueous solution of toxins and a 200 mg, 10 mL Bond Elut C18 cartridge were employed. Modifications of the De la Iglesia et al. [38] protocol for DA were made to adjust it for lipophilic toxins to employ a same method for the 16 compounds. To study the changes, every solution that passed through the cartridge was analyzed using the LC-QqQ/MS system.

The SPE technique depends on multiple variables, which were optimized based on methods described in the literature. The SPE cartridges were conditioned with 20 mL of MeOH and 20 mL of 0.1% FA. The aqueous standard solution (50 mL) was acidified with 0.1% FA to keep DA with a neutral charge for retention. After loading the sample, a washed step was applied with 20 mL of 0.1% FA. Then, three elutions were performed using 3 mL of MeOH:water (1:9, *v*/*v*) 0.2 M ammonium acetate. The results showed that toxins were retained in the C18 phase, even after washing. Moreover, DA appeared only in the first elution, meaning that 3 mL was sufficient to recover it. However, lipophilic toxins appeared in all elutions, so their elution would require a higher percentage of MeOH or a higher concentration of ammonium acetate.

**Figure 1:**
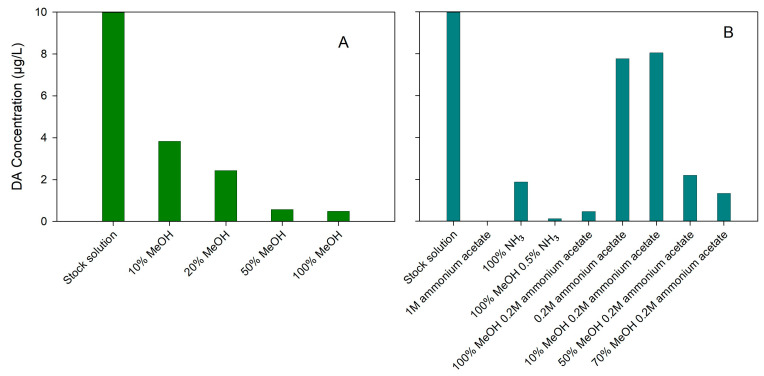
Elution solvents tested in SPE extraction of domoic acid: (**A**) study of methanol percentage; (**B**) study of basic solutions.

**Figure 2:**
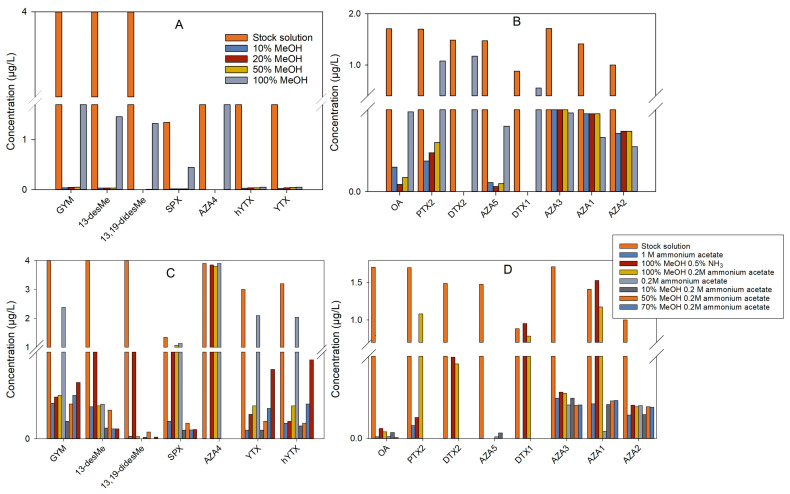
Elution solvents in SPE extraction of lipophilic toxins and yessotoxins tested: (**A**,**B**) study of percentage of methanol; (**C**,**D**) study of basic solutions.

The percentage of MeOH was optimized from 10 to 100, with the recovery of DA decreasing (Figure 1A) and that of lipophilic toxins increasing (Figure 2A,B).

Then, two basic solutions, ammonium acetate and ammonia, were tested at several concentrations and combined with the organic phase. As DA and lipophilic toxins need to be eluted in a different percentage of the organic phase, two elution fractions (E1 and E2) were used. E1 contained 10% MeOH and 0.2 M ammonium acetate, while E2 contained 100% MeOH.

For E1 elution, three volumes (1, 2, and 5 mL) were tested, and the recoveries obtained for DA were 95%, 92%, and 78%, respectively. Therefore, elution E1 was optimized as 1 mL of MeOH:water (1:9, *v*/*v*) 0.2 M ammonium acetate.

Elution E2 was modified with 0.2 M and 1 M of ammonium acetate, and different amounts of NH_3_ (0.25, 0.50, and 1%). The compound signals decrease when the percentage of NH_3_ increases, and the optimal condition is with 2 mL of MeOH and 1 M of ammonium acetate.

Finally, the volume of seawater was studied in the 25–100 mL range. The sample was fortified with toxins at a concentration of 20 ng/mL. Peak areas were larger with larger seawater volumes for some toxins but not for others, so 75 mL was selected because linearity was maintained for all toxins.

### 2.3. Validation of the Measurements

To assure the validity of the measurements, the limits of quantification and detection were calculated and analyses for selectivity, linearity, recovery, and precision were performed [43,44].

Three calibration graphs for every analyte were created using the SPE procedure combined with LC-QqQ-MS/MS by least-squares linear regression analysis of the compound concentrations versus peak area, using five concentration levels. Table 2 presents the analytical characteristics of the optimized protocol, including the slopes obtained from calibration graphs using an aqueous standard solution, natural seawater sample, or synthetic seawater. ANOVA test results showed that there were no statistically significant differences in the slopes, indicating the absence of a matrix effect. Therefore, an aqueous standard addition is recommended for quantification.

The sensitivity of the procedure was evaluated by calculating the limits of quantification (LOQs) and detection (LODs) for a signal-to-noise ratio of 10 and 3, respectively. Table 2 shows that the LODs varied from 0.05 to 91.2 ng L^−1^ for 13,19-didesM and hYTX, respectively.

To determine the repeatability of the method, we calculated the RSD (average relative standard deviation) from 10 replicate analyses of a seawater sample fortified with the analytes at 100 ng L^−1^. The RSD intraday values ranged from 0.44% to 14.9% (Table 2).

The recovery of the SPE procedure was evaluated by fortifying three seawater samples with the analytes at two concentration levels in triplicate (Table 3).

This procedure was applied to the analysis of the toxin contents of 21 seawater samples. There were no interfering peaks at the retention times of the compounds. The analytes were identified by comparing the retention times, their transitions, and the transition ratios between the samples and those obtained from standard solutions. As mentioned above, only DA was found at levels above its LOD.

### 2.4. Domoic Acid and Environmental Variables

**Figure 3:**
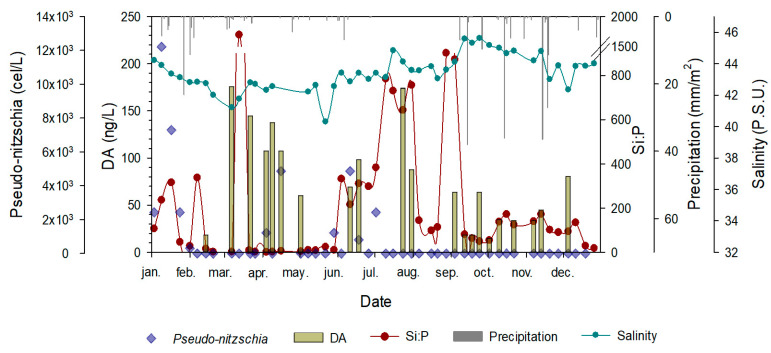
Time series overlap of *Pseudo-nitzschia* density (blue diamonds), domoic acid concentration (pale green bars), silicates to phosphates (Si:P) ratio (red dots and line), precipitation (grey bars from above), and salinity (dark cyan dosts and line) in the Mar Menor lagoon. The water DA concentrations can be explained by a trade-off of bottom-up and top-down factors. Bottom-up factors are characterized by the Si:P ratio as a proxy for nutrients limitation; top-down factors are mainly dilution factor due to precipitation (as a proxy of rain over the lagoon plus run-off of the catchment area) and salinity as a proxy for the exchange of water with the Mediterranean Sea, mainly through the Encañizadas inlet. These factors, together with advection processes, explain the poor correlation between *Pseudo-nitzschia* density and DA concentration.

The evolution of the DA, the only toxin recorded, content in the water is shown in Figure 3 (yellow pale bars), ranging from 1.4 to 224.06 ng L^−1^. The density of all species of the genus *Pseudo-nitzschia* (blue diamonds) ranged from 11 to 9.6·10^6^ cel/L. Finding a good correlation between producer cells and DA is not an easy task due to the many other processes involved as sources of variability. It has been reported that gene expression for DA production varies with environmental conditions [45,46]. For example, some species of *Pseudo-nitzschia* can produce higher levels of DA at salinities in the range found here [33], but also when lacking Fe [47,48], which is not the case as the Mar Menor is highly affected by historical mining waste [49,50], under nitrate from high groundwater discharge [51], under the limitation of Si and P [52,53,54,55], and with a varying pH [56].

The overlapping of the silicates to phosphate molar ratio (Si:P), which is an expression of phosphorous limitation against silicates used by diatoms to form its valves, suggests a positive correlation between Si:P and DA, although more data are required to confirm this hypothesis in our system. Furthermore, the life cycle for *Pseudo-nitzschia* can last from 2–3 days to several weeks [57,58], while the toxin can remain stable in the water for several weeks to a few months [59]. Moreover, not all *Pseudo-nitzschia* species produce the same amount of toxin, and they do not do so under the same exact environmental conditions.

The sediments in the Mar Menor are mostly clay and mud covered with extensive macroalgae meadows of *Caulerpa prolifera* [60,61]. These algae uptake large amounts of nitrogen that, once transformed into biomass, accumulate in the seabed when decomposition of the meadows occurs, mainly in the winter with the low temperatures. Moreover, these meadows act as sediment traps in calm conditions after storms, thus accumulating organic matter in the sediment. Once organic matter decomposes in the anoxic sediment, a large reservoir of ammonia and phosphate is retained in its interstitial waters. Therefore, resuspension is a key process that brings back ammonia and phosphates to the water column, thus fueling phytoplankton growth.

Several pieces of evidence also indicate that DA can accumulate in the sediment [62,63]. If this were the case in the Mar Menor, as it seems to be (Velazquez, pers.com.), resuspension by waves in the very shallow waters would bring this compound back to the water column, thus imposing a larger source for both spatial and temporal variability in the water column.

At the same time, a large spatial variability exists in the phytoplankton of the Mar Menor, which tends to grow forming patches of different densities according to the very local environmental conditions [64], namely nutrients, light field, and turbulence. Advective processes add even more variability to both spatial and temporal scales, being of vital importance in redistributing the phytoplankton patches over the lagoon. As a proxy for this advective process, we have used precipitation (vertical grey bars) and salinity (cyan dots and lines) in Figure 3.

The average annual precipitation in the Mar Menor is 300 mm/year, but much of it falls in torrential downpours over a few days each year. When a precipitation event occurs, the lagoon receives runoff from the water collected in its watershed, which spans 1244 km^2^. Although this freshwater tends to flow to the Mediterranean Sea by sliding over the denser water in the Mar Menor, the short-term effect of rainfall on salinity is significant. The overlap of precipitation with DA evolution over time suggests that precipitation dilutes and removes DA from the water column. Furthermore, the sampling station is located in the area of influence of the Las Encañizadas inlet, which, due to its shallowness and large cross-section, plays a key role in the exchange of water between the lagoon and the Mediterranean Sea [65]. Using salinity at the sampling station as a proxy for water exchange, one can see that several events of large amounts of clean Mediterranean water entering the lagoon cause the salinity to drop, and consequently, DA tends to decrease or even disappear completely, thus reinforcing the idea of the dilution effect as a regulating factor for this toxin in this environment.

## 3. Conclusions

LC-QqQ-MS/MS was applied for the first time to analyze lipophilic, yessotoxins, and domoic acid marine biotoxins in the Mar Menor hypersaline coastal lagoon. The use of this system in combination with the solid phase extraction (SPE) technique allowed for preconcentration at levels at ng L^−1^ and cleaning and desalting of the samples. The determination of the analytes was possible without interferences or matrix effects by employing the multiple reaction monitoring (MRM) mode and standards to determine the specific retention times. This optimized procedure is applicable for monitoring these toxins in hypersaline water.

Although no clear correlation between the concentrations of phytoplankton producers and DA exists due to the many different and large resources of both temporal and spatial variability, data suggests that phosphate limitation against silicates may be a driver for DA in the lagoon. On the other hand, the dilution effect, either by rainfall or by clean water entering the lagoon from the Mediterranean Sea, can play an important role in regulating DA concentrations in the Mar Menor system.

## 4. Material and Methods

### 4.1. Study Area

**Figure 4:**
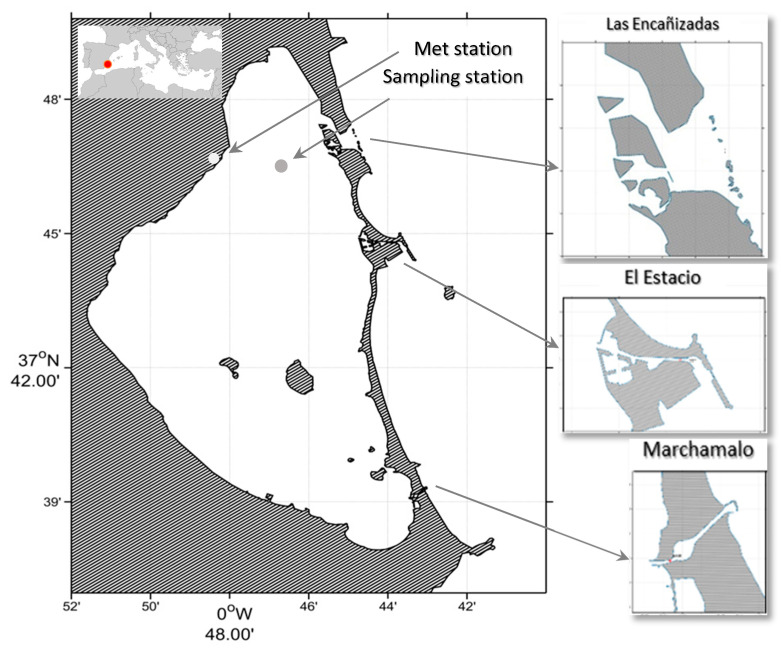
Locations of the Mar Menor coastal lagoon, sampling station, and meteorological station with detail of the three inlets connecting the Mar Menor coastal lagoon with the Mediterranean Sea. The cross-sectional area of the Las Encañizadas inlet is 40% of the total cross-sectional area of the lagoon, which has a heavy influence on the water exchange at the sampling point.

The Mar Menor is a microtidal lagoon located on the southeastern coast of Spain (Figure 4). It covers an area of 175 km^2^, with a maximum depth of 6.5 m (an average of 3.5 m), making it the largest hypersaline lagoon in Europe with a salinity range of 38.5 to 47.5 P.S.U. During the summer season, the highest mean temperature recorded is 32 °C, and the average annual evaporation exceeds precipitation.

Three inlets connect the lagoon with the Mediterranean Sea: Las Encañizadas, El Estacio, and Marchamalo. Las Encañizadas is a complex of channels that can be either dry or wet, depending on the sea water level. It has a cross-sectional area of 163 m^2^ (40% of the lagoon’s total cross-sectional area) and plays a crucial role in the exchange of water during storms. Due to its shallowness, its influence is greater during high water levels, mainly due to atmospheric pressure rather than tides, which are very small in the Mediterranean.

Since 2016, the Mar Menor lagoon has been experiencing severe intermittent eutrophication episodes [66,67] resulting in several anoxic events that have caused mass mortality of fishes in 2019 and 2021. Intensive agriculture in the watershed covering more than 1244 km^2^, urban development around the lagoon with deficiencies in the sewage system, and poor management practices in the groundwater level have resulted in high levels of nitrates and phosphates entering the lagoon. In 2018, a sampling station (Figure 4) in the northern part of the lagoon performed regular sampling throughout the year.

### 4.2. Environmental Variables

Precipitation data were obtained from the Spanish Met Agency (AEMET) station at San Javier Airport (Figure 4). Water temperature and salinity profiles were measured using a CTD (Castaway, Sontek - Xylem, WA, USA). Water samples were collected using a Lund’s tube sampler [68], which integrated the entire water column from surface to bottom. Three sub-samples were collected and filtered through GF/F filters of 25 mm diameter using a syringe, and the filtrate was stored at −24 °C until the analysis of nutrients. Another three subsamples were kept at 4 °C until the analysis for toxins. One more subsample was stored in 250 mL bottles and fixed with acid-lugol until further analysis.

The analysis of nutrients (nitrate, nitrite, ammonium, phosphate, and silicate) was carried out using a high-precision colorimeter on an AA3 segmented flow autoanalyzer (Seal Analytical, Germany) following the [69] method.

To estimate cell abundance and identify taxa, the fixed samples were sedimented in Utermöhl chambers [70] for a minimum of 24 h. The samples were then counted using an inverted microscope (Leica DM-IL) equipped with a 63× magnification objective.

A summary of the environmental variables recorded is given in Table 4.

### 4.3. LC-MS Analytical Method

Analytical methods of marine toxins include in vitro assays, the most widely used method of mouse bioassays and chromatographic methods [71,72], coupled with different detectors, such us fluorescence [37,73] and ultraviolet [39,74], and mass spectrometry (MS) [1,27,34,38,41,42,74].

To obtain an analytical method able to analyze toxins, which are expected to be in a concentration of the order of parts per trillion, it is necessary to apply a preconcentration stage to the samples, such as the SPE technique to eliminate interferences, the seawater matrix effect, and to simultaneously analyze a wide range of concentrations of several toxins.

Other extraction techniques, such as magnetic solid phase extraction (MSPE) [75], use of a molecularly imprinted polymer (MIP) [39,76], dispersive liquid–liquid microextraction (DLLME) [27], and dispersive micro solid phase extraction (DMSPE) [77], allow only the analysis of a small group of toxins.

### 4.4. Chemicals and Materials

High quality acetonitrile, methanol (MeOH) and water of LC-MS quality were obtained from Chem-Lab NV (Zedelgem, Belgium). Analytical standards of 13,19-didesmethyl spirolide C (13,19-didesM), 13-desmethyl spirolide C (13-desM), 20-methyl spirolide G (SPX20G), Azaspiracid 1 (AZA1), Azaspiracid 2 (AZA2), Azaspiracid 3 (AZA3), Azaspiracid 4 (AZA4), Azaspiracid 5 (AZA5), Dinophysistoxin 1 (DTX1), Dinophysistoxin 2 (DTX2), Okadoic acid (OA), Yessotoxin (YTX), and Hommo-Yessotoxin (hYTX) were obtained from Laboratorio Cifga, S.A. (Lugo, Spain). Pectenotoxin 2 (PTX2) and Gymnodimine (GYM) were purchased from National Research Council, Institute for Marine Biosciences (NRC CNRC, Halifax, NS, Canada) and DA form Sigma–Aldrich (St. Louis, MO, USA).

Other reagents that were required, such us formic acid (FA) (98% purity), were provided by Panreac (Barcelona, Spain). Water was deionized through a Milli-Q water purification system (Billerica, MA, USA).

### 4.5. Sampling and Pre-Treatment

Samples were collected in 500 mL capacity polystyrene container and stored at 4 °C. A 75 mL volume sample was filtered using a GF/F filter and acidified with 0.1% FA; the SPE procedure was then applied.

A Visiprep^TM^ SPE Vacuum Manifold from Supelco (Sigma–Aldrich) was used to preconcentrate analytes in Bond Elut LRC C18 SPE cartridges, 200 mg, 10 mL from Agilent Technologies. Bond Elut C18 cartridge was conditioned with 20 mL of MeOH and 20 mL of 0.1% FA. The pretreated sample was passed through the cartridge at a flowrate of 1 mL min^−1^. The cartridge was washed with 20 mL of 0.1% FA and two solutions (E1 and E2) were needed to elute the analytes. Firstly, 1 mL of MeOH:water (1:9, *v*/*v*) and 0.2 M ammonium acetate (E1) preconcentrated the DA, and the lipophilic toxins were eluted with E2 (2 mL of methanol and 1 M ammonium acetate). Both fractions were filtered employing a 0.45 µm nylon filter and were transferred to two LC amber vials and, finally, injected into the LC system.

For recovery studies, three seawater samples were fortified at two concentration levels in triplicate at 5 and 10 ng L^−1^ of AZAs, 20 and 50 of SPX, YTXs, DTX1, and PTX2, 25 and 50 ng L^−1^ of DTX2, 50 and 100 ng L^−1^ of DA, 200 and 400 ng L^−1^ of OA, and 500 and 1000 ng L^−1^ of GYM.

### 4.6. Instrumentation

The analysis was performed using an HPLC Agilent 1260 Infinity system equipped with a quaternary pump (G1311B) and an autosampler (G1329B), with an 8 µL injection volume. The analytical column utilized for the reversed-phase technique was a Zorbax SB-C18 (2.1×75 mm, 3.5 µm) from Agilent Technologies, held in an oven at 30 °C with a flow rate of 0.3 mL/min, operating in gradient mode. For positive ionization mode (ESI+), the mobile phase consisted of a solvent mixture of 2 mM ammonium acetate and 0.1% FA in water (solvent A) and MeOH (solvent B). For negative mode (ESI-), the mobile phase comprised 2 mM ammonium acetate in MeOH (solvent C) and water (solvent D), as described in previous studies [27].

The SPE technique was used with two elution fractions (E1 and E2) to preconcentrate the toxins. The E1 elution was used to recover DA, while lipophilic toxins were extracted in the E2 elution. Since the E1 elution contains only one target compound, a different gradient program is required for each elution. To elute the DA toxin, the gradient program started at 25% B for 3 min, increased to 95% B in 4 min, and was maintained for 4 min, then returned to the initial conditions in 3 min. To separate lipophilic toxins, the gradient program started at 25% B for 2.5 min, increased to 60% B in half a min, and was maintained for 5 min, increased to 75% B and was maintained for 7 min, then the column was cleaned by establishing the percentage of B at 95 for 4 min, and initial conditions were recovered in 30 min.

The E2 elution also contains yessotoxins that are ionized in negative mode, requiring a different gradient program: the initial mobile phase consisted of 70% of C and 30% of D for 2 min, then the percentage of C was increased to 95 in half a minute and was maintained for 5 min, and initial conditions were recovered in 15 min.

The MS used was an Agilent G6410A triple quadrupole. The Agilent Mass Hunter Data Acquisition (Qualitative and Quantitative Analysis) was used for data acquisition and method development. Electrospray source ionization (ESI) was operated in negative mode for YTX and hYTX and in positive ion mode for the remaining compounds. Nitrogen was used as the nebulizer and collision gas at the following conditions: nebulizer pressure 40 psi, capillary voltage 4500 V, drying gas flow 8 L/min, and temperature 350 °C.

To optimize MRM transitions, each compound was injected into the LC-MS/MS-QqQ system at a concentration close to 1 µg/mL. Firstly, mass spectra were set in the 80–1000 amu *m*/*z* range, and the precursor ion was selected. Then, product ions were selected by applying different collision energies (CE). Finally, fragmentor voltages were studied from 70 up to 200 V. The selected conditions are shown in Table 1. The most sensitive transition was used for quantification purposes, and the rest of the MRM transitions were used for the identification of the compounds.

## Figures and Tables

**Table 1:** LC-MS/MS-QqQ parameters.

Compound	MRM Transition (*m*/*z*)	CE (V)	Fragmentor Voltage (V)	Retention Time (min)
DA	312 → 266 Q	10	120	3.4
	312 → 248 q	10		
	312 → 161 q	20		
YTX	1141.5 → 1061.3 Q	35	135	4.7
	1141.5 → 925.5 q	60		
hYTX	1155.4 → 1075.5 Q	35	135	4.7
GYM	508.3 → 490.6 Q	40	200	6.9
13-desM	692 → 164 Q	50	200	7.7
	692 → 444 q	20		
13,19-didesM	678 → 164 Q	50	200	7.3
	678 → 430 q	40		
SPX20G	706 → 688 Q	30	190	8.1
	706 → 670 q	35		
OA	827 → 723 Q	55	190	13.8
	827 → 809 q	45		
DTX2	827 → 723 Q	55	190	14.7
	827 → 809 q	45		
AZA4	844 → 826 Q	30	190	14.7
	844 → 808 q	45		
PTX2	881.5 → 837.5 Q	60	230	14.8
DTX1	841 → 823 Q	45	190	17.5
	841 → 737 q	55		
AZA3	828 → 810 Q	30	190	18.6
	828 → 792 q	45		
AZA5	844 → 826 Q	30	180	19.0
	844 → 808 q	40		
AZA1	842 → 824 Q	30	190	19.4
	842 → 806 q	45		
AZA2	856 → 838 Q	30	190	19.9
	856 → 820 q	45		

Q = quantifier, q = qualifier; Described in [27].

**Table 2:** Analytical characteristics for SPE-LC-QqQ-MS/MS procedure.

Compound	Aqueous Standard Slope (L ng^−1^)	Natural Seawater Slope (L ng^−1^)	Synthetic Seawater Slope (L ng^−1^)	Lineal Range Studied (ng L^−1^)	LOD (ng L^−1^)	LOQ (ng L^−1^)	RSD (%)
DA	5.41	6.29	5.74	45–900	14.8	44.6	6.19
GYM	25.4	20.2	22.8	16–100	35.0	105	2.31
13-desM	469	444	479	20–200	0.06	0.17	10.2
13,19-didesM	564	397	553	20–200	0.05	0.16	10.4
SPX	215	200	256	20–200	0.15	0.44	8.09
OA	0.64	0.69	0.75	200–2000	80.6	242	9.12
PTX2	4.37	5.13	4.14	15–100	2.29	6.86	10.8
AZA4	575	614	807	3–50	0.72	2.16	6.01
DTX2	69.5	66.8	71.4	26–133	8.63	25.9	6.34
AZA5	84.5	158	141	3–50	1.04	3.12	9.19
DTX1	4.91	6.18	5.27	26–133	8.95	26.9	13.6
hYTX	2.31	2.50	2.22	100–1100	91.2	274	12.5
YTX	2.15	2.47	2.07	100–1100	51.7	155	6.34
AZA3	235	247	353	3–50	1.71	5.13	14.9
AZA1	427	595	722	3–50	0.15	0.45	0.44
AZA2	315	408	481	3–50	0.20	0.61	3.36

LOD: limit of detection; LOQ: limit of quantification; RSD: average relative standard deviation.

**Table 3:** SPE procedure recovery study.

Compound	Level Concentration (ng L^−1^)	Recovery (%)
DA	50	83
	100	75
GYM	500	118
	1000	115
13-desM	20	80
	50	79
13,19-didesM	20	82
	50	74
SPX	50	91
	200	80
OA	200	90
	400	107
PTX2	50	119
	200	90
AZA4	5	112
	10	88
DTX2	25	106
	50	107
AZA5	5	110
	10	90
DTX1	50	120
	200	122
hYTX	50	81
	200	77
YTX	50	108
	200	78
AZA3	5	87
	10	105
AZA1	5	99
	10	110
AZA2	5	114
	10	120

**Table 4:** Environmental parameters at the sampling station in the Mar Menor.

	Max	Min	Mean	Std
Temperature (°C)	31	9.9	20.86	6.48
Salinity (PSU)	45.61	39.75	43.06	1.37
Nitrate [NO_3_^−^] (μM)	3.83	0.009	0.43	0.75
Nitrite [NO_2_^−^] (μM)	0.477	0.01	0.1	0.12
Ammonia [NH_4_^+^] (μM)	10.2	0.04	2.38	2.31
Phosphates [PO_4_^−^] (μM)	0.434	0.002	0.14	0.12
Silicates [Si(OH)_4_] (μM)	33.6	0.03	10.58	8.77
DIN	11.001	0.07	2.92	2.55
DIN:P	1872.5	0.389	92.72	267.18
DIN:Si	108.5	0.005	5.85	19.42
Si:P	1690	0.084	221.29	326.99
*Pseudo-nitzschia* (cel/L)	1.20 × 10^7^	0	1.30 × 10^6^	3.00 × 10^6^
DA (ng/L)	224.06	14.84	80.09	56.78

## Data Availability

On request to the corresponding author.
